# Acetabular Edge Loading During Gait Is Elevated by the Anatomical Deformities of Hip Dysplasia

**DOI:** 10.3389/fspor.2021.687419

**Published:** 2021-07-01

**Authors:** Ke Song, Cecilia Pascual-Garrido, John C. Clohisy, Michael D. Harris

**Affiliations:** ^1^Program in Physical Therapy, Movement Science Research Center, Washington University in St. Louis School of Medicine, St. Louis, MO, United States; ^2^Department of Mechanical Engineering and Materials Science, Washington University in St. Louis, St. Louis, MO, United States; ^3^Department of Orthopaedic Surgery, Washington University in St. Louis School of Medicine, St. Louis, MO, United States

**Keywords:** acetabular edge loading, hip dysplasia, labral tears, biomechanics, gait, musculoskeletal modeling, subject-specific

## Abstract

Developmental dysplasia of the hip (DDH) is a known risk factor for articular tissue damage and secondary hip osteoarthritis. Acetabular labral tears are prevalent in hips with DDH and may result from excessive loading at the edge of the shallow acetabulum. Location-specific risks for labral tears may also depend on neuromuscular factors such as movement patterns and muscle-induced hip joint reaction forces (JRFs). To evaluate such mechanically-induced risks, we used subject-specific musculoskeletal models to compare acetabular edge loading (AEL) during gait between individuals with DDH (*N* = 15) and healthy controls (*N* = 15), and determined the associations between AEL and radiographic measures of DDH acetabular anatomy. The three-dimensional pelvis and femur anatomy of each DDH and control subject were reconstructed from magnetic resonance images and used to personalize hip joint center locations and muscle paths in each model. Model-estimated hip JRFs were projected onto the three-dimensional acetabular rim to predict instantaneous AEL forces and their accumulative impulses throughout a gait cycle. Compared to controls, subjects with DDH demonstrated significantly higher AEL in the antero-superior acetabulum during early stance (3.6 vs. 2.8 × BW, *p* ≤ 0.01), late stance (4.3 vs. 3.3 × BW, *p* ≤ 0.05), and throughout the gait cycle (1.8 vs. 1.4 × BW^*^s, *p* ≤ 0.02), despite having similar hip movement patterns. Elevated AEL primarily occurred in regions where the shallow acetabular edge was in close proximity to the hip JRF direction, and was strongly correlated with the radiographic severity of acetabular deformities. The results suggest AEL is highly dependent on movement and muscle-induced joint loading, and significantly elevated by the DDH acetabular deformities.

## Introduction

Developmental dysplasia of the hip (DDH) is most commonly characterized by a shallow acetabulum and is a primary risk factor for pre-mature development of hip osteoarthritis (Gala et al., 2016; Beaulé, 2020). The main catalyst of hip osteoarthritis secondary to DDH is articular tissue damage resulting from aberrant loading (Felson, 2013), especially near the labrum on the lateral edge of acetabulum (Lewis and Sahrmann, 2006; Hartig-Andreasen et al., 2013). Tears to the acetabular labrum are highly prevalent in patients with DDH, often painful, and can limit joint function (Burnett et al., 2006; Hartig-Andreasen et al., 2013; Gala et al., 2016). Such mechanically-induced tears, whether untreated or unresolved after surgery, may then induce detrimental mechano-biological changes that advance hip joint degeneration (Lewis and Sahrmann, 2006; Cvetanovich et al., 2015; Beaulé, 2020).

Effectively assessing or treating mechanically-induced labral tears requires first understanding the major contributors to acetabular edge loading (AEL). Because direct measurement of AEL is not possible, computer simulation of articular loading has been used to study both healthy and dysplastic hips. In DDH, contributions of abnormal or surgically-altered bones to chondro-labral mechanics have both been demonstrated by finite element models with detailed acetabular anatomy (Henak et al., 2014; Abraham et al., 2017). While these prior models provided valuable insights about intra-articular mechanics in hips with DDH, they were driven using generic loading conditions and omitted the influence of two major contributors to AEL, namely subject-specific movement patterns and muscle-induced joint reaction forces (JRFs) (Thomas-Aitken et al., 2018).

The influence of movement and JRFs on articular mechanics may be assessed using dynamic neuromusculoskeletal models (Delp et al., 2007). Musculoskeletal modeling studies have previously been used to estimate AEL following total-hip or resurfacing arthroplasty and have helped quantify the risks for implant wear with various movement patterns or implant positions (Mellon et al., 2013, 2015; van Arkel et al., 2013; Wesseling et al., 2016). Yet to date, musculoskeletal models have not been used to estimate AEL in native hips. A reason for the lack of such studies could be that the generic anatomy used in most models does not closely represent the bony deformities of dysplastic hips (Song et al., 2019), and cannot be used to characterize hip joint loading beyond JRFs. Recently, we showed that image-based musculoskeletal models can delineate joint and muscle mechanical differences, including JRFs, between hips with and without DDH (Harris et al., 2017; Song et al., 2020). Still, because interpreting the clinical meanings of JRFs remains challenging, more precise ways to quantify joint loading and its associations with damage are needed. By combining subject-specific bony anatomy, movement patterns *and* muscle-induced JRFs, image-based models can provide refined AEL quantification and advance our understanding of how these factors collectively contribute to DDH pathomechanics and hip joint degeneration.

In addition to understanding the pathomechanics of DDH, it is important to know how mechanical variables such as AEL relate to clinically measurable variables. The clinical severity of DDH is most commonly assessed using radiographic measures of acetabular anatomy, namely the lateral center-edge angle (LCEA) and acetabular inclination (AI) (Wiberg, 1939; Tönnis, 1987). For hips with DDH, an LCEA < 20° and AI > 10° are considered clinical indicators of structural instability (Clohisy et al., 2008). However, without knowing a clear relationship between standard radiographic measures of DDH used in clinics and lab-based variables of pathomechanics, clinical risk assessment of DDH-related labral tears and articular cartilage damage remains a challenge. Identifying the associations between AEL and structural characteristics such as LCEA and AI can help bridge biomechanical and radiographic evaluation of patients to improve personalized risk assessments of mechanically-induced damage.

Accordingly, the objectives of this study were to (1) use image-based musculoskeletal models to estimate AEL in hips with DDH compared to healthy control hips during gait, and (2) determine the associations between AEL and radiographic measures of DDH acetabular anatomy (LCEA and AI). We hypothesized that AEL during gait would be higher in antero-superior regions of the acetabula with DDH compared to controls, and that AEL magnitude would be associated with the radiographic severity of DDH acetabular deformities.

## Methods

### Subjects and Data Collection

After Institutional Review Board approval and informed consent, 15 female patients with untreated DDH and 15 female healthy control subjects were included, as previously reported (Song et al., 2020). An *a priori* power analysis based on prior hip JRFs findings during gait (Harris et al., 2017) indicated 15 subjects per group could detect inter-group differences with a statistical power of 0.8. Patients were diagnosed by a single orthopedic surgeon (JCC), and had radiographic evidence of an LCEA < 20° (Wiberg, 1939). Twelve of the 15 DDH subjects had bilateral radiographic signs of DDH deformity, but all had unilateral hip or groin pain lasting over 3 months. Control subjects had no self-reported history of hip pathology, no history of groin or lateral hip pain, had no discomfort during a clinical exam of hip flexion-adduction-internal-rotation, and were confirmed to have no evidence of DDH visible on magnetic resonance images. From the magnetic resonance images, as well as plain film radiographs and clinical histories for the patients, subjects in both groups had no indication other hip deformities, including Legg-Calves-Perthes disease avascular necrosis of the femoral head, slipped capital femoral epiphysis, or femoracetabular impingement. Both groups had no past hip or lower extremity surgeries, or functional restraints that would limit gait movements.

**Figure 1:**
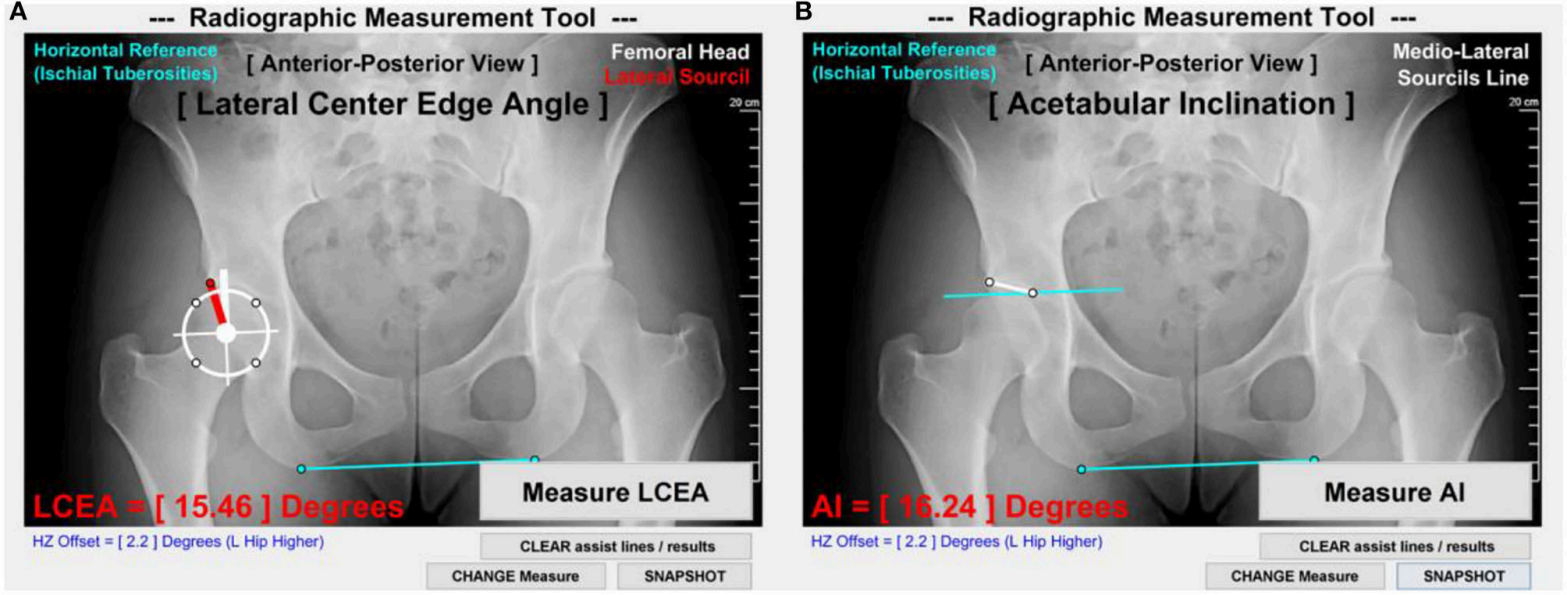
LCEA and AI measurement methods. **(A)** LCEA was measured as the angle between a first line (thick white) through the femoral head center and perpendicular to the inferior aspect of ischial tuberosities (light blue) and a second line connecting the femoral head center to the lateral aspect of acetabular sourcil (red). **(B)** AI was measured as the angle between a first line parallel to the inferior aspect of ischial tuberosities and a second line connecting the medial and lateral aspects of acetabular sourcils (thin white).

On antero-posterior radiographs available for each DDH subject, the LCEA and AI angles were measured following established techniques (Clohisy et al., 2008). The measurements were standardized with a customized Matlab image analysis tool (MathWorks; Natick, MA) (Figure 1) and made by a senior rater with 10 years of experience (MDH) using methods shown to have excellent intra- and inter-rater reliability (Nepple et al., 2014).

With each DDH and control subject lying prone in a neutral hip position, magnetic resonance images were collected from the lumbar region to the knees using a 3T scanner (VIDA, Siemens AG; Munich, Germany) with T1-weighted VIBE gradient-echo sequences and SPAIR fat suppression (1 × 1 × 1 mm voxels) (Song et al., 2020). From the images, 3D bony anatomy of the whole pelvis and femurs was reconstructed using Amira software (v2019a; Thermo Fisher Scientific; Houston, TX), including detailed acetabular anatomy.

Motion data were collected at 100 Hz using 10 infrared cameras (Vicon; Centennial, CO) and 70 skin markers. Each subject first walked three times at a comfortable pace across a 10 m walkway with 2.5 m of additional acceleration and deceleration space. The average speed from the three trials was set as that subject's self-selected speed for treadmill gait. Subjects then walked on an instrumented treadmill (Bertec; Columbus, OH), with a 5-min warmup (Zeni and Higginson, 2010) before three 5-s trials were recorded. Ground reaction forces were recorded at 2,000 Hz. Marker data were low-pass filtered with an 8 Hz cutoff frequency as determined with a residual analysis (Winter, 2009). Force data were filtered at 6 Hz to minimize treadmill analog artifact noise (Pickle et al., 2016).

### Subject-Specific Musculoskeletal Models

Subject-specific musculoskeletal models were created in the OpenSim software (Delp et al., 2007) as recently described (Song et al., 2020). Briefly, a generic OpenSim model (Lai et al., 2017) was modified by adding image-based pelvis and femur bony anatomy, including landmark-based 3D alignment of the pelvis tilt, obliquity, and rotation. Aligned 3D bony anatomy was then used to update hip joint center (HJC) locations, muscle anatomical paths, and muscle-tendon physiological parameters specific to each subject. These models were validated with electromyography as previously reported (Song et al., 2020).

For each subject, kinematic trajectories of the model were qualitatively compared across the three 5-s gait trials. From these trials, one gait cycle representative of the subject's movement was chosen for simulation in OpenSim to estimate time-dependent hip biomechanics. Hip joint angles and net moments were calculated via inverse kinematics and inverse dynamics (Winter, 2009). Hip resultant JRFs and antero-posterior, supero-inferior, and medio-lateral JRF components were computed using OpenSim Joint Reaction Analysis (Steele et al., 2012), using muscle forces estimated via static optimization that minimized the sum-square of muscle activations (Wesseling et al., 2015). Hip JRF components were expressed in the pelvis coordinate system to represent loading onto the acetabulum. JRFs, joint angles and moments on the symptomatic side of each DDH subject were chosen for subsequent analyses; for comparison, a random hip was chosen for each control subject.

### Estimation of Acetabular Edge Loading (AEL)

**Figure 2:**
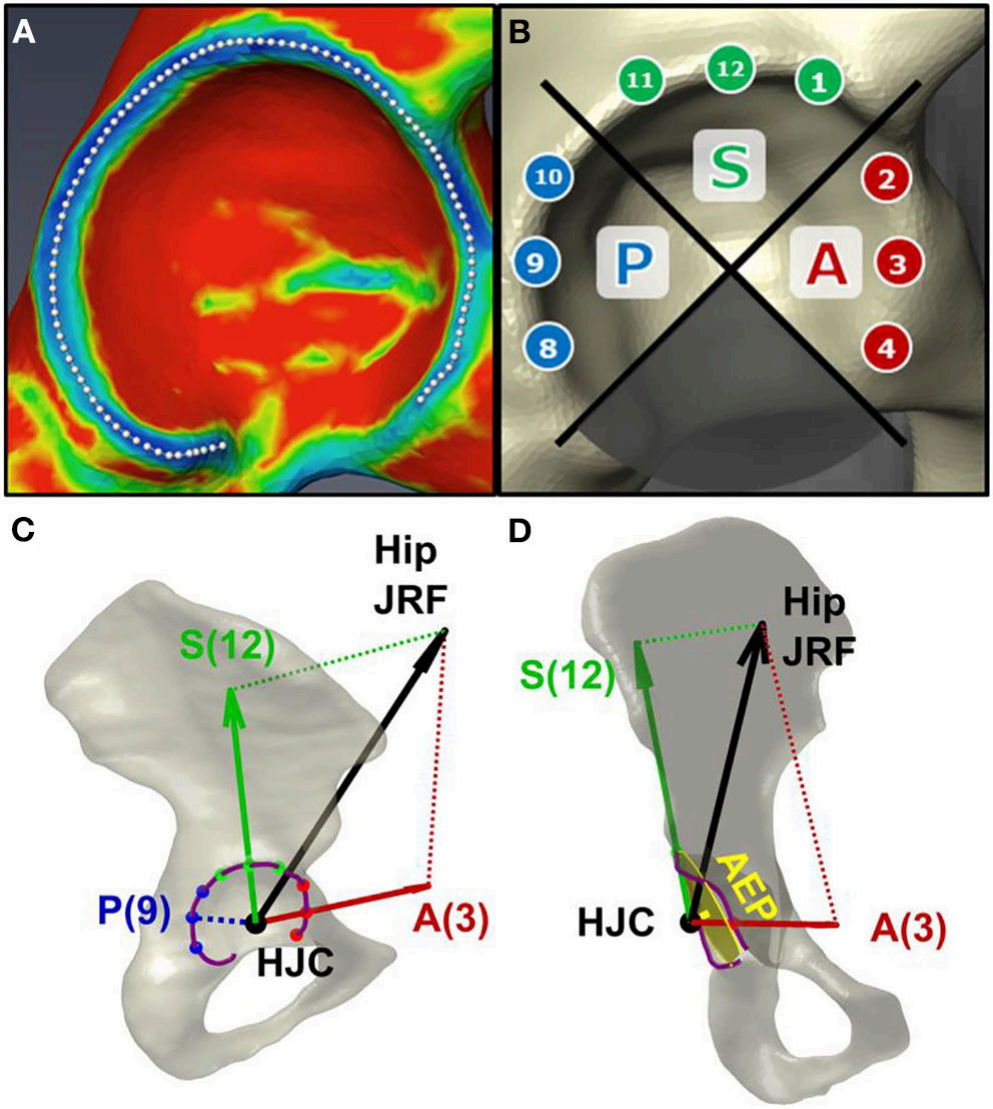
Estimation of acetabular edge loading (AEL). **(A)** The acetabular rim of each subject was delineated using a principal curvature heat map. **(B)** Nine clock-face points were designated on the anterior (“A”), superior (“S”), and posterior (“P”) quadrants of the rim. **(C)** AEL magnitudes were estimated via trigonometric projection of the hip JRF (black arrow) along the directions from HJC toward each clock-face point on the rim (red/green arrows). The JRF-to-edge angle was calculated as the angle between the JRF and the AEL directions (i.e., between black and red/green arrows). Note zero posterior AEL when JRF is directed anteriorly. **(D)** An “acetabular edge plane (AEP)” was fit to the rim to measure the distance between the approximated acetabular border and the HJC.

AEL on the analyzed hip during each gait trial was computed by mathematically projecting hip JRFs onto the acetabular anatomy in each subject-specific model. First, the acetabular rim was delineated on each image-based 3D pelvis, using a principle curvature heat map (Figure 2A). Then, on each acetabular rim, nine clock-face points were designated within the anterior (2-4 o'clock), superior (11–1 o'clock), and posterior (8–10 o'clock) quadrants (Goronzy et al., 2019) (Figure 2B). A right-view clock-face convention was adopted for all hips regardless of side such that 3 o'clock represented anterior for both right and left hips (Goronzy et al., 2019).

The hip JRF was represented as a 3D force vector stemming from the femoral head, i.e., the HJC (Figure 2C). The direction of AEL was defined as the vector from HJC to a point on the acetabular rim. The AEL magnitude was then estimated via trigonometric projection of the JRF along the AEL direction toward each of the 9 clock-face points (Figure 2C). Additionally, a “JRF-to-edge angle” was defined as the angle between the JRF direction and the AEL direction, which represented how close the JRF was relative to the edge (Wesseling et al., 2016). The JRF-to-edge angle was also computed at each clock-face point.

Because the JRF magnitude and direction change during gait, the clock-face AEL magnitude and JRF-to-edge angle are both time-dependent, and were calculated at each time frame throughout the gait trial. AEL was then numerically integrated over the duration of the whole gait cycle to calculate its accumulative impulse.

Finally, a 3D plane was fit to each delineated acetabular rim, termed the “acetabular edge plane (AEP)” (Figure 2D). The distance from each HJC to AEP was calculated to approximate the relative position between the femoral head center and the acetabular border, as an additional measure of the DDH anatomical deformity.

### Inter-group Comparison and Correlations

Hip JRFs, clock-face AEL, and JRF-to-edge angles were time-normalized to 0–100% of a gait cycle. The forces were then normalized by body weight (unit: × BW). To include the influence of the gait cycle duration, the accumulative impulses of AEL were not time-normalized, but magnitudes were normalized by BW (unit: × BW^*^s). Net hip moments were normalized by body mass (unit: Nm/kg). Timing of the two hip JRF peaks in early stance (termed “JRF1”) and late stance (“JRF2”) in each gait cycle was identified. All instantaneous forces, angles, and moments at the times of JRF1 and JRF2 were extracted for statistical analyses, along with the accumulative impulses.

Each demographic, radiographic, and biomechanical variable was assessed for normality using the Shapiro-Wilk test. Normally distributed variables were compared between the DDH and control groups using independent *t*-tests, with corrections for heterogeneity of variance as needed. Variables violating data normality were compared using the non-parametric Mann-Whitney *U* tests. Statistical significance for all tests was α = 0.05. Effect sizes were determined by Cohen's *d*, with a large effect defined as *d* ≥ 0.8 (Cohen, 1988). Within the DDH subjects, associations between biomechanical variables (JRFs, AEL, JRF-to-edge angles) and radiographic measures (LCEA and AI) were assessed using Pearson's correlation (*r*), or Spearman's rank correlation (ρ) if data violated normality; a strong correlation was defined as |*r*| or |ρ| ≥ 0.5 (Cohen, 1988).

## Results

### Subject Demographics and Anatomy

The DDH and control groups did not differ significantly in age, height, mass, body-mass index, or gait speed (Table 1). The average LCEA and AI values for the DDH group were within ranges of traditional DDH definitions (Clohisy et al., 2008). Additionally, the HJC-to-AEP distance was significantly larger in hips with DDH compared to controls (Table 1), which strongly correlated with smaller LCEA (ρ = −0.53) and larger AI (*r* = 0.58) among the DDH subjects.

**Table 1:** Demographics, gait speed, radiographic measures, and the HJC-to-AEP distance (mean ± SD) of DDH and control subjects.

	**DDH (*N* = 15)**	**Control (*N* = 15)**	***P*-value**
Age (years)	26.5 ± 7.9	24.6 ± 6.3	0.62
Height (m)	1.66 ± 0.07	1.67 ± 0.06	0.85
Mass (kg)	62.7 ± 9.3	61.9 ± 7.8	0.79
Body-mass index (kg/m^2^)	22.7 ± 2.4	22.3 ± 2.3	0.64
Gait speed (m/s)	1.37 ± 0.15	1.39 ± 0.15	0.59
Lateral Center-Edge Angle (degrees)	10.5 ± 9.2	N/A	**-**
Acetabular Inclination (degrees)	18.0 ± 8.4	N/A	**-**
HJC-to-AEP distance (mm)	9.3 ± 2.5	5.9 ± 1.4	<0.01

*Radiographic measurements of acetabular anatomy were only made for the DDH subjects. HJC, hip joint center; AEP, acetabular edge plane*.

### Hip JRFs

As reported in our previous study (Song et al., 2020), DDH subjects had higher-than-control medial hip JRFs at JRF1 (1.3 ± 0.6 vs. 0.9 ± 0.3 × BW; *p* = 0.03, *d* = 0.82), as well as higher resultant (5.7 ± 1.1 vs. 5.0 ± 0.8 × BW) and superior JRFs (4.8 ± 0.8 vs. 4.1 ± 0.7 × BW) at JRF2 (*p* ≤ 0.05, *d* ≥ 0.76).

### Clock-Face AEL and JRF-to-Edge Angles

**Figure 3:**
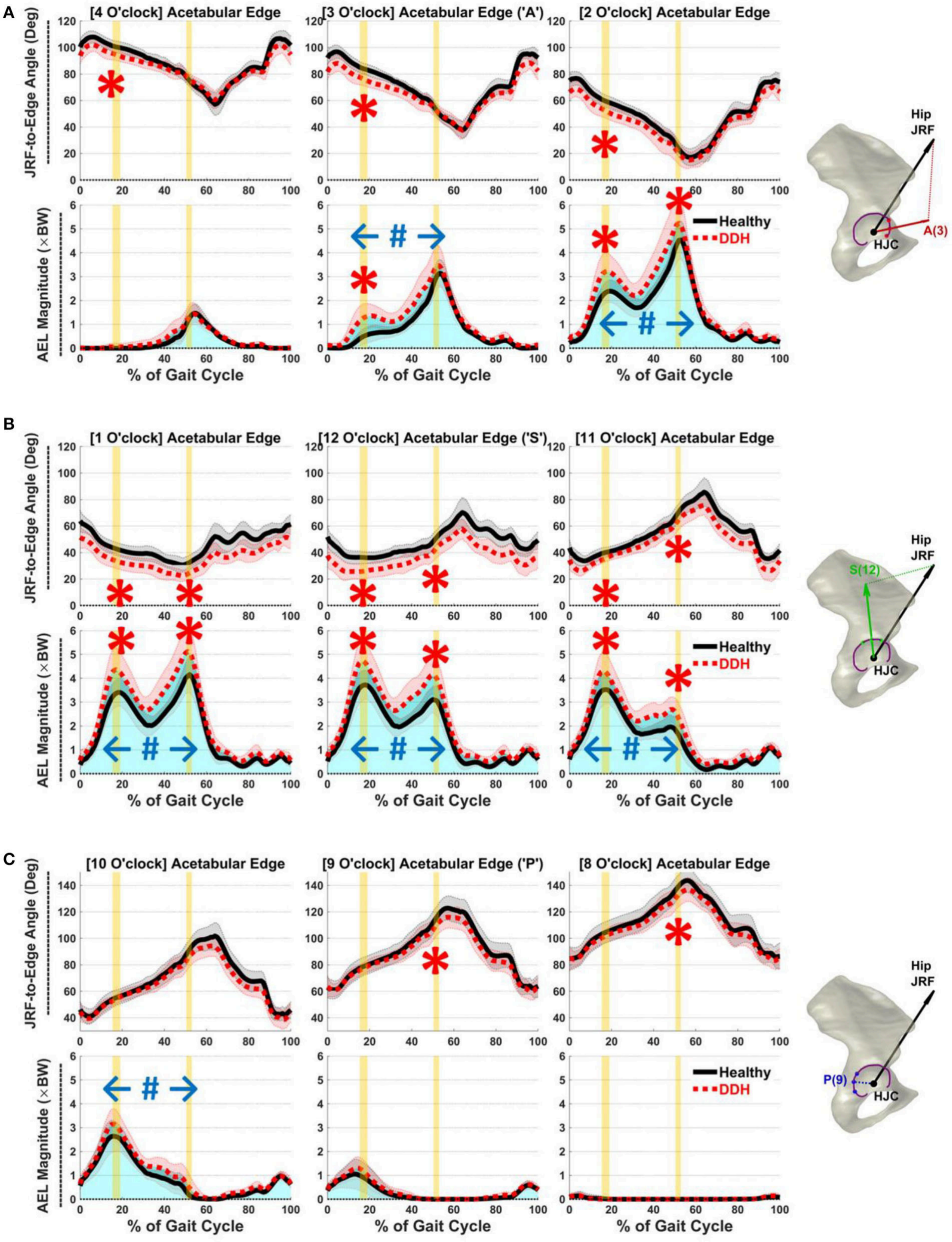
Average JRF-to-edge angles (top) and AEL (bottom) in **(A)** anterior (2–4 o'clock), **(B)** superior (11–1 o'clock), and **(C)** posterior (8–10 o'clock) regions throughout gait. Red/black shades = ±1 SD. Vertical yellow bars indicate time of JRF peaks (JRF1 and JRF2). Blue shades (total area under the curves) illustrate accumulative impulses. Statistical significance: “*” instantaneous, “#” accumulative.

At early-stance JRF1, DDH subjects had higher AEL than controls in the anterior and superior regions from 11 to 3 o'clock (*p* ≤ 0.01, *d* ≥ 0.97; Figures 3A,B). Averaged AEL across the 11 to 3 o'clock points was 3.6 × BW in DDH vs. 2.8 × BW in controls. Higher AEL correlated with smaller LCEA (ρ = −0.58) and larger AI (*r* = 0.53) for DDH subjects at the 3 o'clock location, but not from 11 to 2 o'clock. Simultaneously, JRF-to-edge angles were smaller in hips with DDH in the anterior and superior regions (11–4 o'clock, Figures 3A,B; *p* ≤ 0.01, *d* ≥ 1.18), which correlated with smaller LCEA (ρ ≥ 0.60) and larger AI (*r* ≤ −0.45) from 12 to 3 o'clock.

At late-stance JRF2, similar to early-stance, DDH subjects had higher AEL in the anterior and superior regions from 11 to 2 o'clock (*p* ≤ 0.05, *d* ≥ 0.76; Figures 3A,B), which correlated with larger AI at 11 o'clock (*r* = 0.60). Averaged AEL across the 11 to 2 o'clock points was 4.3 × BW in DDH vs. 3.3 × BW in controls. The JRF-to-edge angles at JRF2 were again smaller in DDH subjects across the superior region (11–1 o'clock, Figure 3B; *p* ≤ 0.02, *d* ≥ 0.92). Posterior AEL magnitudes at JRF2 were minimal, but JRF-to-edge angles were significantly smaller in DDH subjects than controls in the posterior region (8–9 o'clock, Figure 3C; *p* = 0.04, *d* ≥ 0.79). Smaller JRF-to-edge angles correlated with larger AI in all regions (*r* ≤ −0.52), and with smaller LCEA at 1 o'clock (ρ = 0.53).

Over a whole gait cycle, DDH subjects had higher accumulative AEL (i.e., impulse) in a broad region from the anterior to postero-superior acetabulum (10–3 o'clock, Figure 3; *p* ≤ 0.02, *d* ≥ 0.91). Averaged AEL accumulative impulse across the 10 to 3 o'clock points was 1.8 × BW^*^s in DDH vs. 1.4 × BW^*^s in controls. Higher accumulative AEL correlated with smaller LCEA (ρ = −0.54) and larger AI (*r* = 0.51) at the 12 o'clock location.

### Hip Joint Angles and Moments

Also as previously reported (Song et al., 2020), DDH subjects demonstrated slight hip adduction while controls had slight hip abduction (1.2°±2.8° vs.−1.4°±2.6°, *p* = 0.01, *d* = 0.95) compared to controls at the time of JRF2 in late stance (Supplementary Figure 1). Hip moments did not differ between groups (Supplementary Figure 1).

## Discussion

The objectives of this study were to estimate AEL in hips with DDH compared to healthy control hips during gait, and determine the associations between AEL and radiographic measures of DDH acetabular anatomy. Results generally supported the hypothesized AEL elevation in hips with DDH. Our secondary hypothesis that AEL elevation was associated with the severity of acetabular deformities was also supported. Hips with DDH exhibited higher AEL both instantaneously when JRFs peaked, and accumulatively over the duration of gait. The specific location and timing of elevated AEL varied throughout different phases of gait, suggesting relationships among acetabular anatomy, movement, muscle-induced joint loading, and labral mechanics. Such dependencies support the need to comprehensively evaluate the whole hip biomechanical environment for a refined understanding of DDH pathomechanics, and patient-specific risk assessments of DDH-related labral tears and articular cartilage damage.

The severity of acetabular bony deformity was a main contributor to AEL. First, elevated AEL almost always accompanied reduced JRF-to-edge angles, which meant that whenever hip loading acted in close proximity to the shallow acetabular edge, a large component of the JRF would be projected to the edge. This coupled phenomenon was consistent in the anterior and superior regions of the DDH acetabula, which matches well-established clinical descriptions of the locations where DDH-related labral tears frequently occur (Hartig-Andreasen et al., 2013). Prior models of articular cartilage stress found that the bony deformities of DDH led to a disproportionately large amount of contact stresses on the superolateral labrum (Henak et al., 2014). Our results support this edge loading phenomenon, and provide new evidence of how the shallow acetabulum of DDH also causes muscle-induced edge loading to be elevated. We also found that the shallower the acetabulum was, as shown by LCEA and AI, the higher JRF loading would be applied at the lateral edge in late stance and over a gait cycle. Additionally, a larger HJC-to-AEP distance demonstrated that the lateral edge of dysplastic hips was farther away from the femoral head center than controls (Gala et al., 2016; Cheng et al., 2019), which further elevated AEL. Based on these associations, region-specific risks for labral tears or cartilage damage can vary according to radiographic metrics of acetabular deformity and in context with muscle-induced pathomechanics.

Labral tears can be caused by both acute and chronic mechanisms (Lewis and Sahrmann, 2006). High acute hip loading during gait typically occurs in a transient phase of motion, such as weight acceptance during early stance (i.e., JRF1) and the late-stance transition to push-off (i.e., JRF2). Hip loading from JRFs is generally in the supero-medial direction throughout a gait cycle, and shifts from posterior to anterior over stance (Bergmann et al., 2001; Lewis et al., 2010). We found that instantaneous AEL was elevated in hips with DDH at both JRF1 and JRF2. Cyclic high instantaneous loading on the superior and anterior acetabulum when JRFs peak may be another risk factor that compounds with the shallow acetabulum to heighten the likelihood of labral tears and articular cartilage damage in those regions (Lewis and Sahrmann, 2006; Gala et al., 2016). Although high instantaneous loads can occur during traumatic events, a large percentage of labral tears cannot be linked to known high-impact events (Santori and Villar, 2000; Burnett et al., 2006). Instead, most tears may be caused by accrued micro-damage from routine yet aberrant loading (McCarthy et al., 2001). Muscle-induced AEL may contribute to such insidious damage not only at cyclic points when JRFs peak, but also through accumulative loads across the entire gait motion. Indeed, accumulative AEL during gait was not only significantly increased in our patients with DDH, but also spanned a wide region around the acetabular rim. Because the duration of abnormal AEL could play a vital role in the development of labral tears, the assessments of labral mechanics in response to disease progression or treatments should be monitored over time.

It is notable that antero-superior AEL was elevated not just in late stance when anterior JRFs peaked, but also in early stance when the hip was flexed and the joint loading was less anterior. While JRFs were directed farther away from anterior edge in both groups during early stance (Figure 3A), the JRF-to-edge angles were *relatively* smaller in DDH subjects vs. controls. Such inter-group differences may explain why AEL was *relatively* elevated and can be caused by the DDH subjects' higher medial JRFs during early stance. Although medial loading may be produced by the hip muscles to stabilize the femoral head in the shallow acetabulum (Harris et al., 2017), due to the dynamic nature of hip loading and 3D acetabular positions, a force component may still be projected toward the shallow anterior edge. This dynamic interaction may also explain why the lateral acetabular anatomy (LCEA and AI) was associated with an anterior AEL in early stance. Its potential contributions to labral and cartilage damage should not be overlooked, especially considering the accumulative impacts (Figure 3).

Several limitations of this study should be considered. First, due to a small sample size, it was not feasible to statistically analyze the interactions between AEL and the different subgroups of posterior, anterior, and global acetabular deficiency (Nepple et al., 2017). We also did not include other radiographic measures beyond LCEA and AI as we chose to focus on the most standard clinical characteristics of the DDH bony anatomy. DDH patients with poor anterior and posterior femoral coverage may possess different risks of edge loading and labral tears, and respond differently to peak or repetitive loading during movements. While we reported AEL for the DDH group as a whole, our methods were precise to individuals, which could be readily applied to subgroup analyses given a large enough sample, as well as the relationships between AEL and other measures of the hip anatomy beyond LCEA and AI. Also, because radiographs were not available for the healthy controls, analyses of relationships between AEL and radiographic measures were limited the patients. To further confirm the relationships between AEL and hip bony anatomy, such analyses can be extended to radiographic measures of the healthy hips when available. A second limitation was that HJC locations in the musculoskeletal models were assumed static within the acetabulum. Due to the potential instability of dysplastic hips (Beaulé, 2020), subtle translation of the femoral head during motion may occur and could affect projected AEL. However, by defining JRF and AEL directions both stemming from the HJC, their relative closeness (i.e., JRF-to-edge angle) should still robustly capture the mechanical influence of the acetabular deformities. Third, we used static optimization to estimate muscle forces, JRFs and AEL, which did not incorporate muscle co-contractions that could be altered in hips with DDH. We chose this method as it was able to estimate hip JRFs during gait close to benchmark data (Wesseling et al., 2015). To study high-speed movements that involve significant muscle co-contractions, dynamic force estimation may be necessary.

In conclusion, AEL was significantly elevated in hips with DDH compared to healthy controls, both instantaneously when JRFs peaked and accumulatively over the duration of gait. The extent of high AEL was strongly correlated with the severity of DDH deformities, especially lateral acetabular deficiency. Our findings suggest that AEL magnitude and location are highly dependent on movement and muscle-induced joint loading, and significantly elevated by the DDH acetabular deformities.

## Data Availability Statement

The raw data supporting the conclusions of this article will be made available by the authors, without undue reservation.

## Ethics Statement

The studies involving human participants were reviewed and approved by Washington University in St Louis Institutional Review Board. Written informed consent to participate in this study was provided by the participants' legal guardian/next of kin.

## Author Contributions

KS, CP-G, JC, and MH conceptualized the study. MH secured the funding support (see Funding). KS and MH designed the methods, collected and processed image and motion data, with assistance from laboratory research assistants (see Acknowledgments). KS, JC, and MH processed radiographic data. KS created the musculoskeletal models, performed data analyses, and wrote the original article draft. All authors interpreted the data, reviewed and revised the article, and approved the submitted version.

## Conflict of Interest

The authors declare that the research was conducted in the absence of any commercial or financial relationships that could be construed as a potential conflict of interest.
