# Supplementary figures and images for: Acetabular Edge Loading During Gait Is Elevated by the Anatomical Deformities of Hip Dysplasia
Source: Front Sports Act Living. 2021 Jul 1;3:687419. doi: 10.3389/fspor.2021.687419 (PMC8281296; doi:10.3389/fspor.2021.687419)

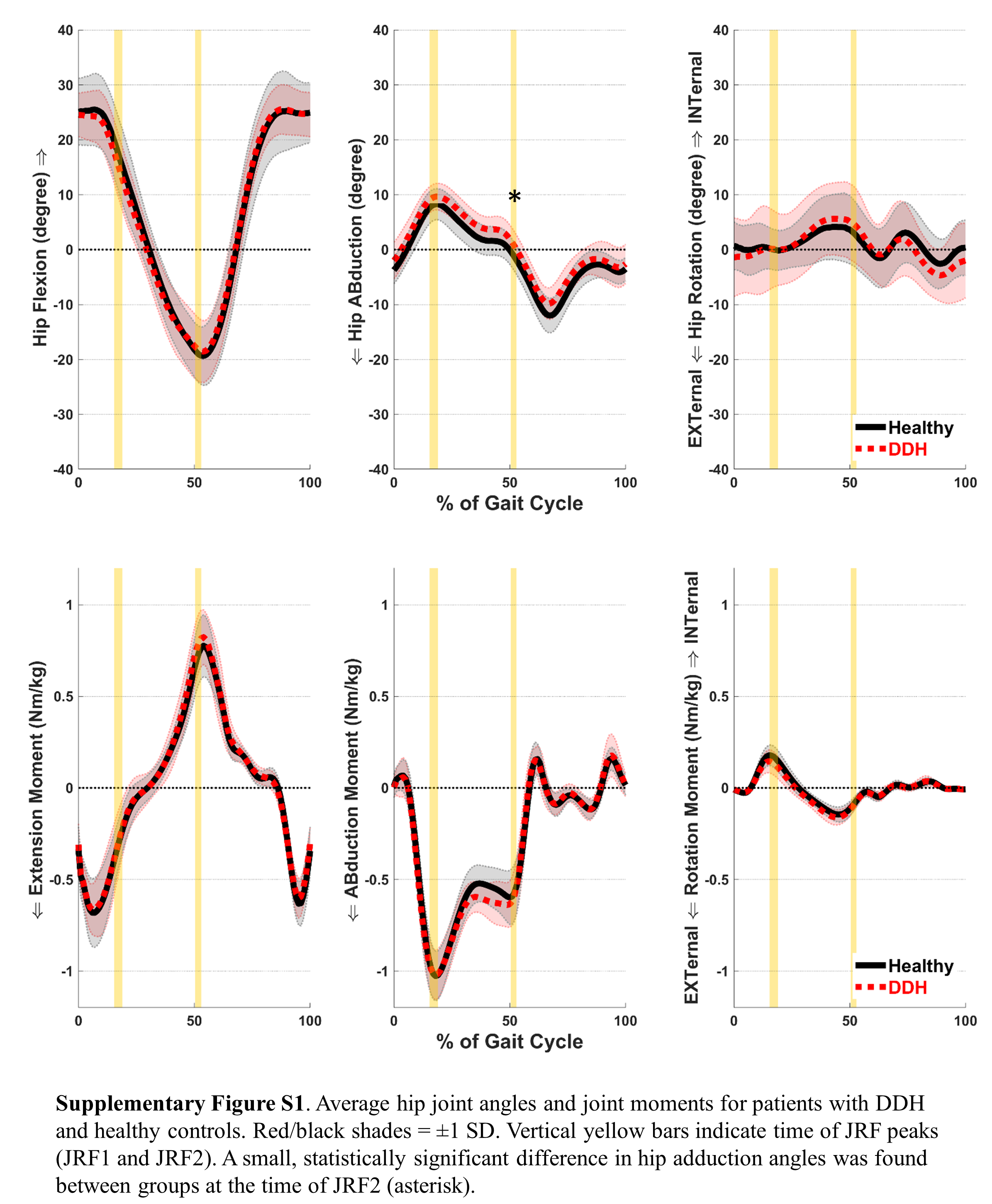

Supplement: Supplementary file 1 [file Image_1.TIF]
